# Supplementary material for: Whole genome sequencing identifies genetic variants associated with neurogenic inflammation in rosacea
Source: Nat Commun. 2023 Jul 5;14:3958. doi: 10.1038/s41467-023-39761-2 (PMC10319783; doi:10.1038/s41467-023-39761-2)
Supplement: Supplementary file 1 — Supplementary Information [file 41467_2023_39761_MOESM1_ESM.pdf]

Supplementary Information

Whole genome sequencing identifies genetic variants associated with neurogenic inflammation in rosacea

Zhili Deng[1, 2, 3, #], Mengting Chen[1, 2, 3, #], Zhixiang Zhao[1, 2, 3], Wenqin Xiao[1, 2, 3], Tangxiele Liu[1, 2, 3], Qinqin Peng[1, 2, 3], Zheng Wu[1, 2, 3], San Xu[1, 2, 3], Wei Shi[1, 2, 3], Dan Jian[1, 2, 3], Ben Wang[1, 2, 3], Fangfen Liu[1, 2, 3], Yan Tang[1, 2, 3], Yingxue Huang[1, 2, 3], Yiya Zhang[1, 2, 3], Qian Wang[4], Lunquan Sun[3, 5], Hongfu Xie[1, 2, 3], Guohong Zhang[6, *], Ji Li[1, 2, 3, *]

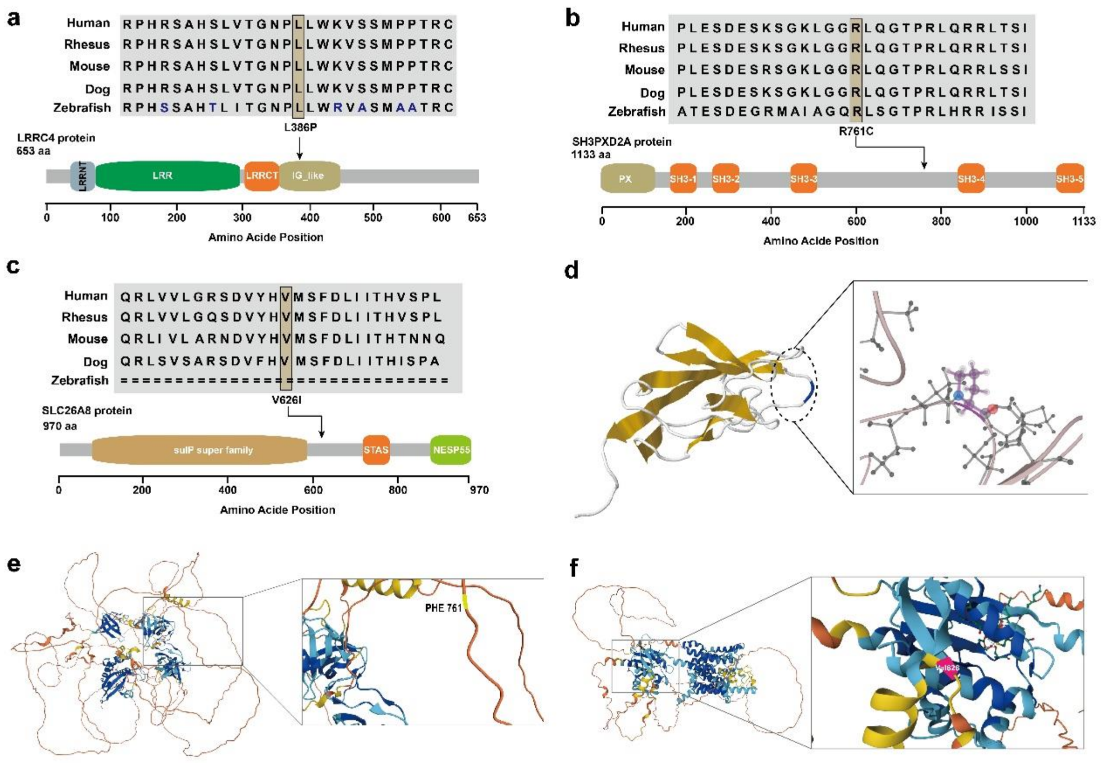

Supplementary Figure 1. Genes with single rare deleterious variants are identified in large rosacea families. a-c Multi-species alignment of conservation of variants in *LRRC4* (**a**), *SH3PXD2A* (**b**) and *SLC26A8* (**c**) genes. The conservation of the mutated amino acids is indicated by the alignment of five species. Evolutionarily conserved positions for nominated pathogenic variants are highlighted in yellow. **d-f** The positions of mutated amino acids in the protein structures of *LRRC4* (**d**), *SH3PXD2A* (**e**)，and *SLC26A8* (**f**).

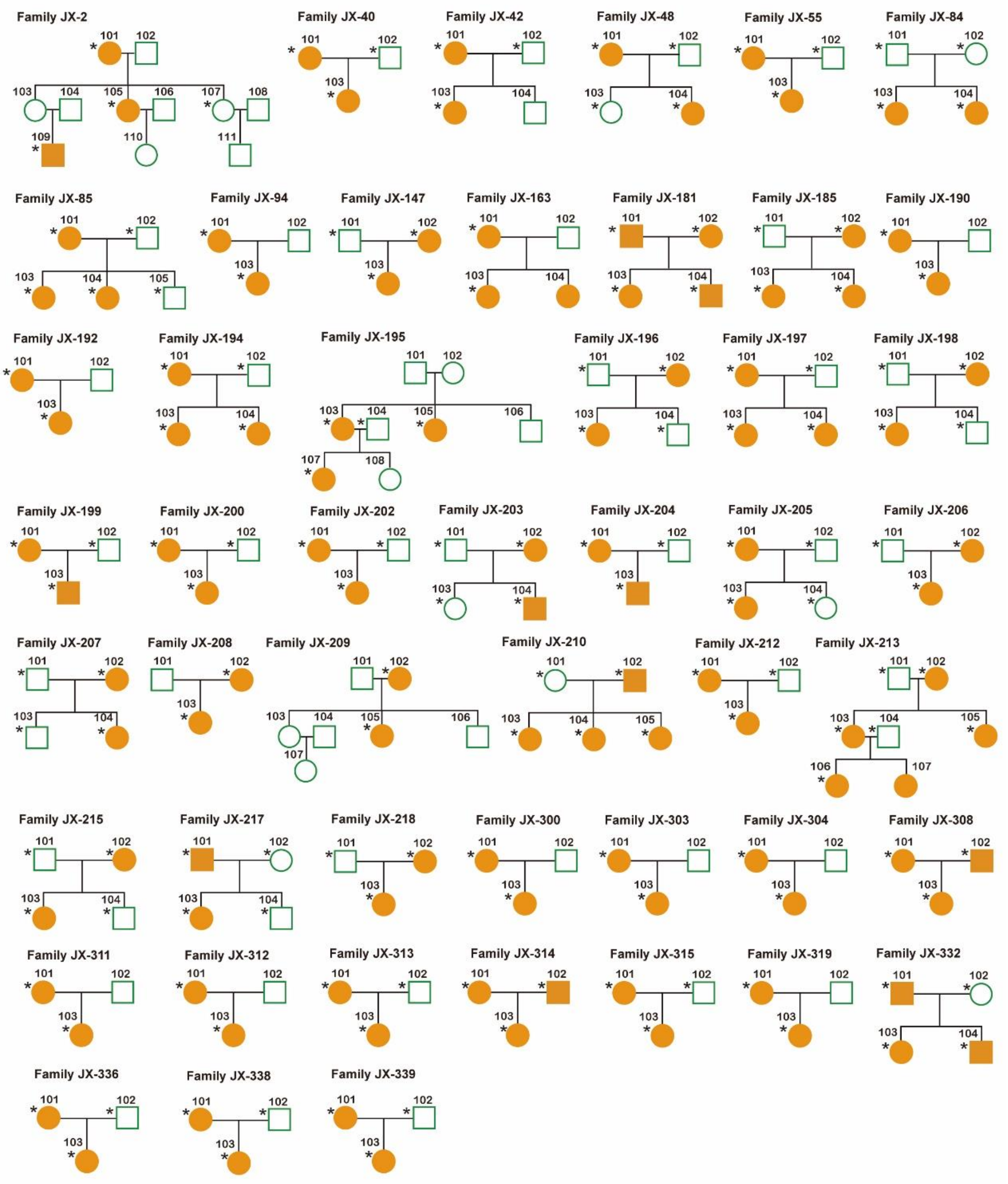

Supplementary Figure 2. Pedigree structures of 49 small rosacea families. Solid symbols indicate individuals affected with rosacea; open symbols denote unaffected relatives; squares indicate male individuals; circles denote female individuals. Individuals with whole exome sequencing are indicated by single asterisks.

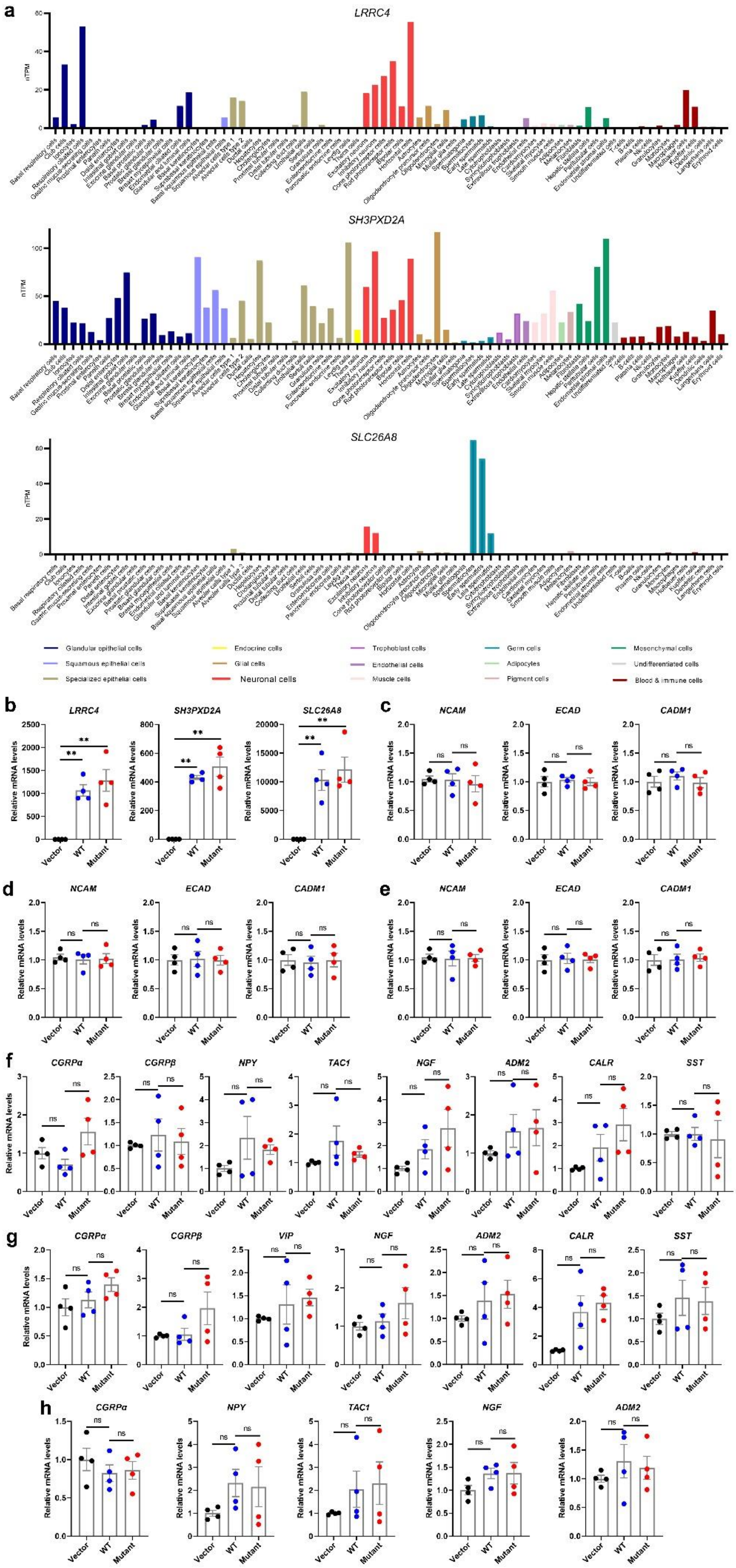
a
LRRC4
SH3PXD2A
SLC26A8
nTPM
Glandular epithelial cells
Squamous epithelial cells
Specialized epithelial cells
Endocrine cells
Glial cells
Neuronal cells
Trophoblast cells
Endothelial cells
Muscle cells
Germ cells
Adipocytes
Pigment cells
Mesenchymal cells
Undifferentiated cells
Blood & immune cells
b
c
d
e
f
g
h
NCAM
ECAD
CADM1
CGRPα
CGRPβ
NPY
TAC1
NGF
ADM2
CALR
SST
VIP
Relative mRNA levels
Vector
WT
Mutant
**
ns

Supplementary Figure 3. *LRRC4/SH3PXD2A/SLC26A8* Mutation upregulates vasoactive neuropeptides in human neural cells. a The mRNA levels of *LRRC4/SH3PXD2A/SLC26A8* in different cell types of the whole human body analyzed by single cell RNA-sequencing data. Red pillars indicate neural cells. **b** The relative mRNA levels of *LRRC4/SH3PXD2A/SLC26A8* in human neural cells transfected respectively with *LRRC4, SH3PXD2A* and *SLC26A8* mutant/WT/control vector plasmids (n = 4 biologically independent experiments). *LRRC4*: Mutant vs Vector, P = 0.0007; WT vs Vector, P = 0.0027, *SH3PXD2A*: Mutant vs Vector, $P < 0.0001$; WT vs Vector, $P < 0.0001$, *SLC26A8*: Mutant vs Vector, P = 0.0016; WT vs Vector, P = 0.0047. **c-e** The relative mRNA levels of *NCAM, ECAD* and *CADM1* in human neural cells transfected respectively with *LRRC4* (**c**), *SH3PXD2A* (**d**) and *SLC26A8* (**e**) mutant/WT/control vector plasmids (n = 4 biologically independent experiments). **f-h** The relative mRNA levels of *CGRPα, CGRPβ, VIP, NPY, TAC1, NGF, ADM2, CALR* and *SST* in human neural cells transfected respectively with *LRRC4* (**f**), *SH3PXD2A* (**g**) and *SLC26A8* (**h**) mutant/WT/control vector plasmids (n = 4 biologically independent experiments). Data represent the mean ± SEM. **P < 0.01. ns indicates no significance. one-way ANOVA with Bonferroni's post hoc test was used.

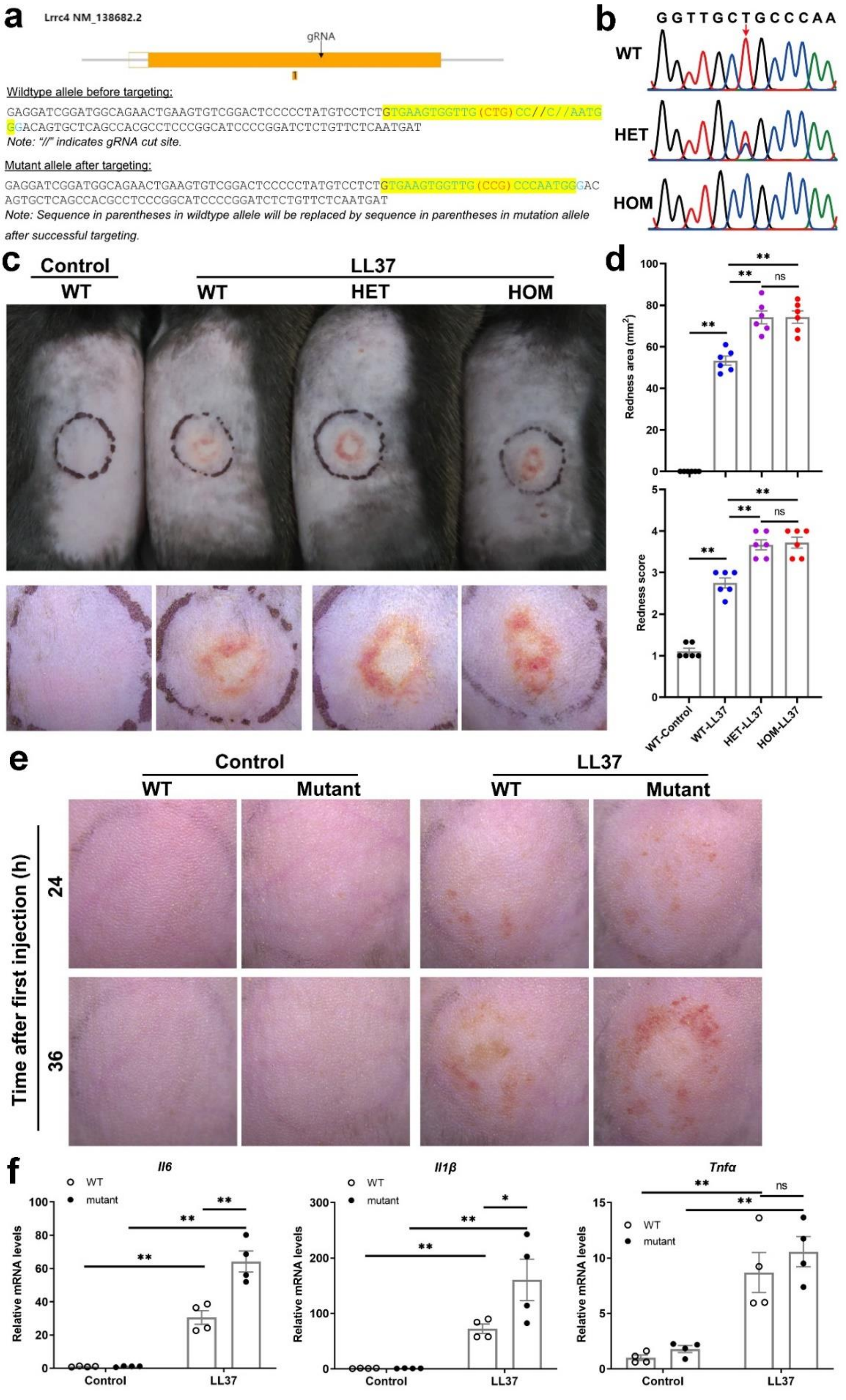

Supplementary Figure 4. *Lrrc4* mutation aggravates rosacea development in mice.

a Schematic illustration of knock-in strategy of *Lrrc4* mutation mice harboring L385P mutation. **b** The genotypes of WT, heterozygotes (HET) and homozygotes (HOM) mice

were validated by sanger DNA sequencing. **c** The back skins of WT, HET and HOM mice intradermally injected with LL37 or control vehicle. Images were taken 48 h after the first LL37 injection. Below panels, magnified images of black dotted circle areas. **d** The severity of the rosacea-like features after first LL37 injection for 48 h, was evaluated with the redness area and score (n = 6 for each group). Redness area: WT-LL37 vs WT-Control, $P < 0.0001$; HET-LL37 vs WT-LL37, $P < 0.0001$; HOM-LL37 vs WT-LL37, $P < 0.0001$; HOM-LL37 vs HET-LL37, $P > 0.9999$. Redness score: WT-LL37 vs WT-Control, $P < 0.0001$; HET-LL37 vs WT-LL37, $P < 0.0001$; HOM-LL37 vs WT-LL37, $P < 0.0001$; HOM-LL37 vs HET-LL37, $P = 0.9997$. **e** The back skins of WT and *Lrrc4* mutant mice intradermally injected with LL37 or control vehicle. Images were taken 24 h and 36h after the first LL37 injection. **f** The relative mRNA levels of *Il6, Il1β* and *Tnfα* in lesional skins from WT and *Lrrc4* mutant mice intradermally injected with LL37 or control vehicle (n = 4 for each group). *Il6:* Mutant (LL37) vs WT (LL37), $P = 0.0002$; Mutant (LL37) vs Mutant (Control), $P < 0.0001$; WT (LL37) vs WT (Control), $P = 0.0007$. *Il1β:* Mutant (LL37) vs WT (LL37), $P = 0.0307$; Mutant (LL37) vs Mutant (Control), $P = 0.0004$; WT (LL37) vs WT (Control), $P < 0.0001$. *Tnfα:* Mutant (LL37) vs WT (LL37), $P = 0.6655$; Mutant (LL37) vs Mutant (Control), $P = 0.0008$; WT (LL37) vs WT (Control), $P = 0.0023$. All results are representative of at least 3 independent experiments. Data represent the mean ± SEM. *P < 0.05, **P < 0.01. ns indicates no significance. one-way ANOVA with Bonferroni's post hoc test was used.

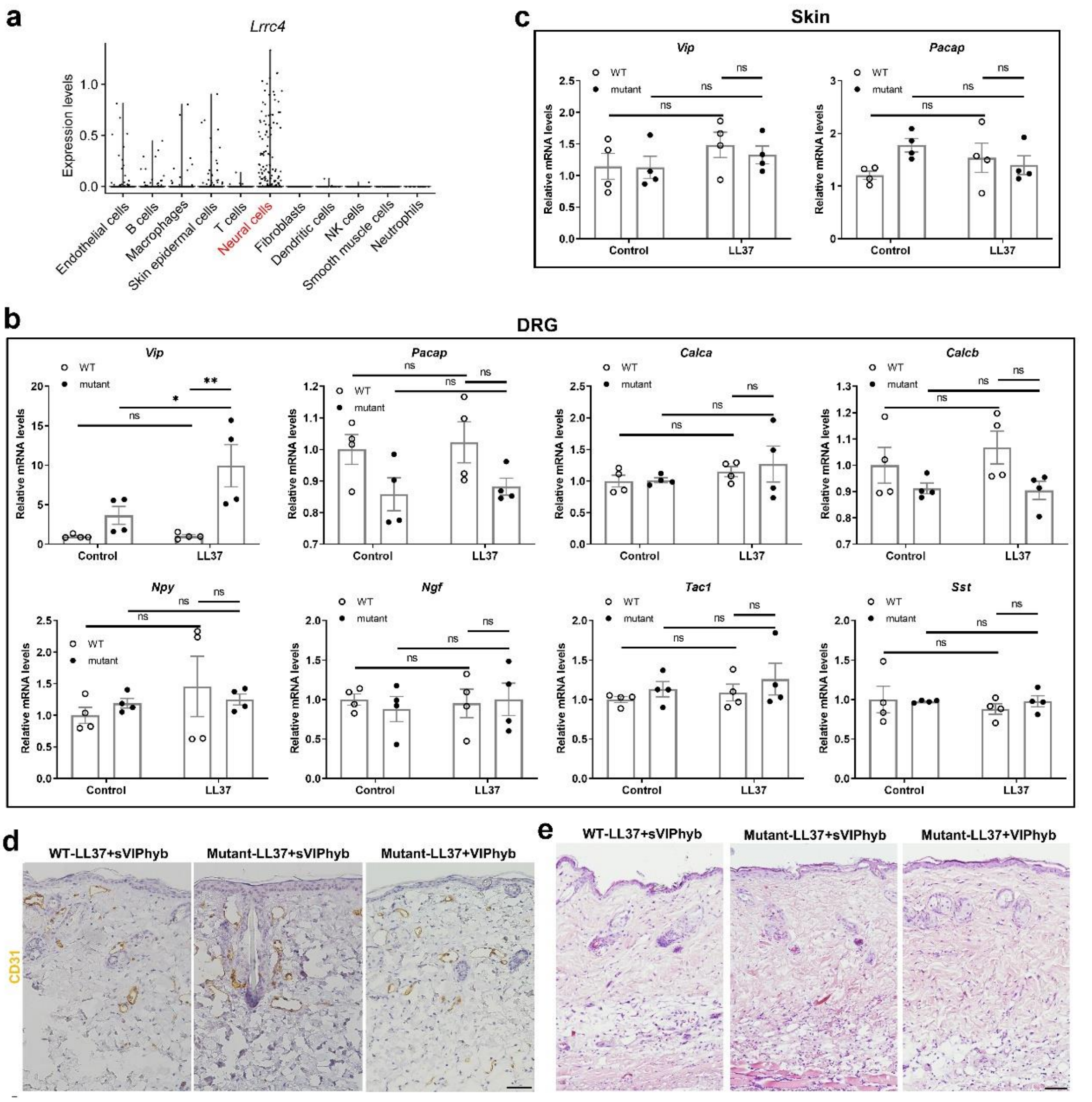

Supplementary Figure 5. Inhibition of VIP signaling improves rosacea-like phenotypes in *Lrrc4* mutat mice. a Violin plots showing the distribution of *Lrrc4* expression in the indicated cell types which may affect skin conditions from young mice (3 months). Skin epidermal cells (n = 636 cells), fibroblasts (n = 129 cells), endothelial cells (n = 3327 cells), neuronal cells (n = 410 cells), T cells (n = 714 cells), B cells (n = 3896 cells), Macrophages (n = 659 cells), NK cells (n = 337 cells), dendritic cells (n = 63 cells), neutrophils (n = 33 cells) and smooth muscle cells (n = 65 cells) were retrieved from Tabula Muris Senis (obtained from the Gene-Expression Omnibus, GSE149590). **b** The relative mRNA levels of *Vip, Pacap, Calca, Calcb, Npy, Ngf, Tac1, Sst* and *Calr* in dorsal root ganglions (DRGs) from WT and mutant mice treated with LL37 or control vehicle (n = 4 mice for each group). *Vip:* Mutant (LL37) vs WT (LL37), P = 0.0047; Mutant (LL37) vs Mutant (Control), P = 0.043; WT (LL37) vs WT (Control), P > 0.9999. *Pacap:* Mutant (LL37)

vs WT (LL37), *P* = 0.2404; Mutant (LL37) vs Mutant (Control), *P* = 0.9858; WT (LL37) vs WT (Control), *P* = 0.9876. *Calca:* Mutant (LL37) vs WT (LL37), *P* = 0.9478; Mutant (LL37) vs Mutant (Control), *P* = 0.6604; WT (LL37) vs WT (Control), *P* = 0.9008. *Calcb:* Mutant (LL37) vs WT (LL37), *P* = 0.1536; Mutant (LL37) vs Mutant (Control), *P* = 0.9995; WT (LL37) vs WT (Control), *P* = 0.7801. *Npy:* Mutant (LL37) vs WT (LL37), *P* = 0.9372; Mutant (LL37) vs Mutant (Control), *P* = 0.9984; WT (LL37) vs WT (Control), *P* = 0.5944. *Ngf:* Mutant (LL37) vs WT (LL37), *P* = 0.9959; Mutant (LL37) vs Mutant (Control), *P* = 0.9483; WT (LL37) vs WT (Control), *P* = 0.9965. *Tac1:* Mutant (LL37) vs WT (LL37), *P* = 0.7729; Mutant (LL37) vs Mutant (Control), *P* = 0.8855; WT (LL37) vs WT (Control), *P* = 0.9568. *Sst:* Mutant (LL37) vs WT (LL37), *P* = 0.8917; Mutant (LL37) vs Mutant (Control), *P* > 0.9999; WT (LL37) vs WT (Control), *P* = 0.8213. **c** The relative mRNA levels of *Vip, Pacap* in skin lesions from WT and mutant mice treated with LL37 or control vehicle (n = 4 mice for each group). **d** IHC of CD31 on skin sections from LL37-treated WT and *Lrrc4* mutant mice intradermally injected with VIPhyb or sVIPhyp. Scale bar, 50 μm. **e** HE staining of lesional skin sections from LL37-treated WT and *Lrrc4* mutant mice intradermally injected with VIPhyb or sVIPhyp. Scale bar, 50 μm. All results are representative of at least 3 independent experiments. Data represent the mean ± SEM. *P < 0.05, **P < 0.01. ns indicates no significance. one-way ANOVA with Bonferroni's post hoc test was used.

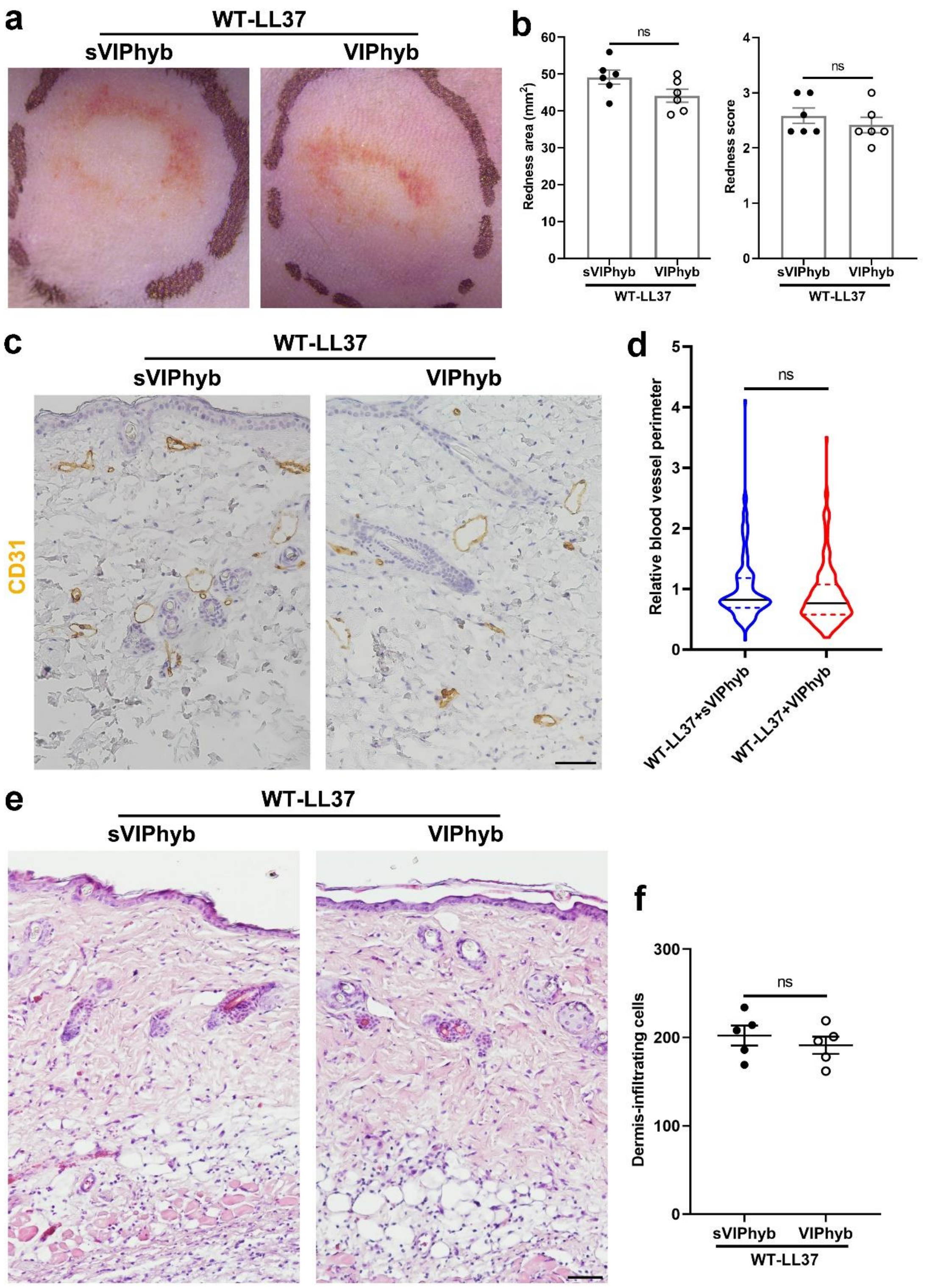

Supplementary Figure 6. VIPhyb injections do not affect the rosacea-like phenotypes induced by LL37 in WT mice. a The back skins of LL37-injected WT mice treated with VIPhyb or sVIPhyb. Images were taken 48 h after the first LL37 injection. **b** The severity of the rosacea-like features after first LL37 injection for 48 h, was evaluated with the redness area and score (n = 6 for each group). **c** IHC of CD31 on skin sections

from LL37-treated WT mice intradermally injected with VIPhyb or sVIPhyp. Scale bar, 50 μm. **d** Quantification of relative blood vessel perimeter in the corresponding groups presented with violin plot. n = 179–182 blood vessels from 5 independent mice for each group. *P* = 0.0701. **e** HE staining of lesional skin sections from LL37-treated WT mice intradermally injected with VIPhyb or sVIPhyp. Scale bar, 50 μm. **f** Dermal infiltrating cells were quantified (n = 5 for each group). *P* = 0.4828. All results are representative of at least 3 independent experiments. Data represent the mean ± SEM. ns indicates no significance. Two-tailed unpaired Student's t-test was used.
