# Supplementary material for: Whole genome sequencing identifies genetic variants associated with neurogenic inflammation in rosacea
Source: Nat Commun. 2023 Jul 5;14:3958. doi: 10.1038/s41467-023-39761-2 (PMC10319783; doi:10.1038/s41467-023-39761-2)
Supplement: Supplementary file 3 — Description of Additional Supplementary Files [file 41467_2023_39761_MOESM3_ESM.pdf]

Description of Additional Supplementary Files

Supplementary Data 1: List of candidate variants identified for each large family by whole-genome sequencing.

Supplementary Data 2: List of candidate variants identified for each small family by whole-exome sequencing.

Supplementary Data 3: DEGs for mutant DGRs vs WT DRGs.

Supplementary Data 4: Detailed information of each subject in rosacea families.

Supplementary Data 5: qPCR primers.
