# Supplementary material for: Whole genome sequencing identifies genetic variants associated with neurogenic inflammation in rosacea
Source: Nat Commun. 2023 Jul 5;14:3958. doi: 10.1038/s41467-023-39761-2 (PMC10319783; doi:10.1038/s41467-023-39761-2)
Supplement: Supplementary file 9 — Reporting Summary [file 41467_2023_39761_MOESM9_ESM.pdf]

nature portfolio

Corresponding author(s): Ji Li, and Guohong Zhang

Last updated by author(s): Jun 16, 2023

Reporting Summary

Nature Portfolio wishes to improve the reproducibility of the work that we publish. This form provides structure for consistency and transparency in reporting. For further information on Nature Portfolio policies, see our Editorial Policies and the Editorial Policy Checklist.

Statistics

For all statistical analyses, confirm that the following items are present in the figure legend, table legend, main text, or Methods section.

n/a	Confirmed	
☐	☒	The exact sample size (*n*) for each experimental group/condition, given as a discrete number and unit of measurement
☐	☒	A statement on whether measurements were taken from distinct samples or whether the same sample was measured repeatedly
☐	☒	The statistical test(s) used AND whether they are one- or two-sided<br>*Only common tests should be described solely by name; describe more complex techniques in the Methods section.*
☐	☒	A description of all covariates tested
☐	☒	A description of any assumptions or corrections, such as tests of normality and adjustment for multiple comparisons
☐	☒	A full description of the statistical parameters including central tendency (e.g. means) or other basic estimates (e.g. regression coefficient) AND variation (e.g. standard deviation) or associated estimates of uncertainty (e.g. confidence intervals)
☐	☒	For null hypothesis testing, the test statistic (e.g. *F*, *t*, *r*) with confidence intervals, effect sizes, degrees of freedom and *P* value noted<br>*Give P values as exact values whenever suitable.*
☒	☐	For Bayesian analysis, information on the choice of priors and Markov chain Monte Carlo settings
☒	☐	For hierarchical and complex designs, identification of the appropriate level for tests and full reporting of outcomes
☒	☐	Estimates of effect sizes (e.g. Cohen's *d*, Pearson's *r*), indicating how they were calculated

Our web collection on statistics for biologists contains articles on many of the points above.

Software and code

Policy information about availability of computer code

Data collection	No software was used for data collection.
Data analysis	Sequencing reads of WGS and WES were mapped to the human reference genome (GRCh38, hg38) with BWA-MEM (v0.7.8).<br>Quality scores were re-aligned with the Indel Realigner algorithm (GATK v3.8.0).<br>In silico functional analysis, SIFT, PolyPhen-2 (v2), CADD (v1.5) and GERP++ (version 2) were used to predict the impact of each nonsynonymous variant on protein function.<br>The Seurat (v4.0) was used as the main tool for single-cell sequencing analysis in R (4.0.3).<br>The ImageJ 1.53 was used for image analysis if immunostaining.<br>Data were processed with GraphPad Prism 8.<br>Details are described in Methods.<br>The codes of our study were present in GitHub (https://github.com/nanlandetian/SkinAging).

For manuscripts utilizing custom algorithms or software that are central to the research but not yet described in published literature, software must be made available to editors and reviewers. We strongly encourage code deposition in a community repository (e.g. GitHub). See the Nature Portfolio guidelines for submitting code & software for further information.

Data

Policy information about availability of data

All manuscripts must include a data availability statement. This statement should provide the following information, where applicable:

- Accession codes, unique identifiers, or web links for publicly available datasets
- A description of any restrictions on data availability
- For clinical datasets or third party data, please ensure that the statement adheres to our policy

All data needed to assess the conclusions in the present study are provided in the manuscript and/or the Supplementary Materials. The uploading and sharing of the genetic data of participated individuals in this project is not permissible in terms of a review by the Human Genetic Resources Administration of China based on regulations documented in the Interim Measures for the Administration of Human Genetic Resources. We have listed the summaries of the mutation data as detailed as possible, and these details are available to other researchers, including the exonic and splicing mutations in large and small rosacea families (shown in Supplementary Data 1 and Supplementary Data 2, respectively). scRNA-seq datasets of different cell types in the human body were downloaded from the Human Protein Atlas database (https://www.proteinatlas.org). scRNA-seq data from the Tabula Muris Senis atlas were obtained from the Gene-Expression Omnibus (accession number : GSE149590). The codes of our study were present in GitHub (https://github.com/nanlandetian/SkinAging). Sequencing data from DRGs of Lrrc4 mutant and WT mice have been deposited in the genome sequence archive under accession number CRA009850 (https://ngdc.cncb.ac.cn/gsa/). The Human reference (GRCh38) dataset required for analysis is avaliable at https://www.ncbi.nlm.nih.gov/assembly/GCF_000001405.26/. Other data supporting the findings of the present study are required to contact with the corresponding author (Ji Li, Email: liji_xy@csu.edu.cn) for identity verification purposes under adhering to the Chinese regulations. Source data are provided with this paper.

Human research participants

Policy information about studies involving human research participants and Sex and Gender in Research.

Reporting on sex and gender	We provided information of gender in Supplementary Table 4. Totally, we collected information from 154 female individuals and 99 male individuals, and 11 skin tissues from 11 female individuals.
Population characteristics	We collected the age, sex, and other clinical information of all individuals in Supplementary Table 4.
Recruitment	Full descriptions of the recruitment and design of each contributing study can be found in the Methods. There are no any self-selection bias or other biases.
Ethics oversight	This study was approved by the ethical committee of the Xiangya Hospital of Central South University.

Note that full information on the approval of the study protocol must also be provided in the manuscript.

Field-specific reporting

Please select the one below that is the best fit for your research. If you are not sure, read the appropriate sections before making your selection.

☒ Life sciences ☐ Behavioural & social sciences ☐ Ecological, evolutionary & environmental sciences

For a reference copy of the document with all sections, see nature.com/documents/nr-reporting-summary-flat.pdf

Life sciences study design

All studies must disclose on these points even when the disclosure is negative.

Sample size	Sample sizes were chosen based on accepted standards in the field and previously published (PMID: 31848144, 34648325, 36470882, or 35768505). For each quantification condition, n≥ 4, and all results are representative of at least 3 independent experiments. No statistical method was used to predetermine the sample size.
Data exclusions	No data were excluded from the analyses.
Replication	All experiments are replicated for at least 3 times, and data are representative of at least 3 independent experiments.
Randomization	Randomization was no relevant to our study. For each familial individuals, the allocation is dependent on phenotype.
Blinding	For human samples, blinding was not relevant because there was no intervention with participants. For mouse and cell experiments, investigators were blinded to all grouping information in data collection and analysis.

Reporting for specific materials, systems and methods

We require information from authors about some types of materials, experimental systems and methods used in many studies. Here, indicate whether each material, system or method listed is relevant to your study. If you are not sure if a list item applies to your research, read the appropriate section before selecting a response.

Materials & experimental systems

n/a	Involved in the study
☐	☒ Antibodies
☐	☒ Eukaryotic cell lines
☒	☐ Palaeontology and archaeology
☐	☒ Animals and other organisms
☒	☐ Clinical data
☒	☐ Dual use research of concern

Methods

n/a	Involved in the study
☒	☐ ChIP-seq
☒	☐ Flow cytometry
☒	☐ MRI-based neuroimaging

Antibodies

Antibodies used

Antibody (dilutions, catalog number, Supplier name)
PACAP (1:200, ab181205, abcam)
VIP (1:200, ab272726, abcam)
CD31 (1:100, 77699, Cell signaling)
PGP9.5 (1:200, ab108986, abcam)

The second antibody used in this study: Alexa Fluor 594-conjugated secondary antibody (1:1000, A-21207, ThermoFisher).

Validation

All antibodies are commercially available and their manufactures provided their validation documents. They were validated for IF or IHC.
https://www.abcam.cn/products/primary-antibodies/pacap-antibody-epr11544-ab181205.html
https://www.abcam.cn/products/primary-antibodies/vip-antibody-epr23288-43-ab272726.html
https://www.cellsignal.com/products/primary-antibodies/cd31-pecam-1-d8v9e-xp-rabbit-mab/77699
https://www.abcam.cn/products/primary-antibodies/pgp95-antibody-epr4118-neuronal-marker-ab108986.html

Eukaryotic cell lines

Policy information about cell lines and Sex and Gender in Research

Cell line source(s)	HCN-2 cells (Cat#CRL-10742) were obtained from the American Type Culture Collection.
Authentication	None of the cells have been authenticated.
Mycoplasma contamination	Cell lines were not tested for mycoplasma contamination but no indication of contamination was observed.
Commonly misidentified lines (See ICLAC register)	No commonly misidentified cell lines were used.

Animals and other research organisms

Policy information about studies involving animals; ARRIVE guidelines recommended for reporting animal research, and Sex and Gender in Research

Laboratory animals	Lrrc4 L385P mutation (generated by Cyagen Biosciences, China) female and male mice of 8 weeks were keeped in specific pathogen-free conditions with a regular 12h light/12 dark cycle, at approximately 20-25 °C and 45-55% humidity.
Wild animals	No wild animals were used in the study.
Reporting on sex	Sex was not considered in this study.
Field-collected samples	No filed collected samples were used in this study.
Ethics oversight	The experiments performed were according to the instructions and permissions of the ethical committee of the Xiangya Hospital of Central South University. This statement was also provided in the manuscript.

Note that full information on the approval of the study protocol must also be provided in the manuscript.
